# SARS-CoV-2 Infection and Rates of Neonatal Congenital Anomalies

**DOI:** 10.1001/jamanetworkopen.2026.11440

**Published:** 2026-05-07

**Authors:** John W. Snelgrove, Rinku Sutradhar, Nancy N. Baxter, Karl Everett, Stephanie C. Lapinsky, Douglas M. Campbell, Mark H. Yudin, Howard Berger, Eliane M. Shore, Andrea N. Simpson

**Affiliations:** 1 Department of Obstetrics and Gynaecology, Temerty Faculty of Medicine, University of Toronto, Toronto, Ontario, Canada; 2 Department of Obstetrics and Gynaecology, Mount Sinai Hospital, Toronto, Ontario, Canada; 3 Institute of Health Policy, Management and Evaluation, Dalla Lana School of Public Health, University of Toronto, Toronto, Ontario, Canada; 4 ICES, Toronto, Ontario, Canada; 5 Department of Surgery, Temerty Faculty of Medicine, University of Toronto, Toronto, Ontario, Canada; 6 Li Ka Shing Knowledge Institute, St Michael’s Hospital, Unity Health Toronto, Toronto, Ontario, Canada; 7 Faculty of Medicine and Health, University of Sydney, Sydney, New South Wales, Australia; 8 Department of Pediatrics, Temerty Faculty of Medicine, University of Toronto, Toronto, Ontario, Canada; 9 Department of Pediatrics, St Michael’s Hospital, Unity Health Toronto, Toronto, Ontario, Canada; 10 Department of Obstetrics and Gynecology, St Michael’s Hospital, Unity Health Toronto, Toronto, Ontario, Canada

## Abstract

**Importance:**

There is conflicting evidence for associations between maternal SARS-CoV-2 infection and neonatal congenital anomalies. Population-based studies evaluating confirmed maternal infection during pregnancy and at specific gestational time periods are needed.

**Objective:**

To evaluate associations between laboratory-confirmed maternal SARS-CoV-2 infection in pregnancy and neonatal congenital anomalies, overall during pregnancy and by specific trimester of exposure.

**Design, Setting, and Participants:**

This population-based, matched cohort study of live births with maternal SARS-CoV-2 infection in pregnancy matched 1:4 to live births without maternal infection in Ontario, Canada, was conducted from December 14, 2020, to December 31, 2021. Matching was performed on maternal age, delivery date, gestational age at birth, neonatal sex, and prepregnancy diabetes. Analyses were conducted from May to August 2025.

**Exposure:**

Maternal SARS-CoV-2 infection in pregnancy confirmed by positive real-time polymerase chain reaction (RT-PCR) test. Pregnancies with no positive RT-PCR test were considered SARS-CoV-2 negative.

**Main Outcomes and Measures:**

The primary outcome was any neonatal congenital anomaly. The secondary outcome was any neonatal cardiac anomaly. Crude incidence rates of congenital anomalies per 1000 live births by maternal SARS-CoV-2 infection status with 95% CI were determined with a Poisson distribution for the study sample overall, and by each trimester of exposure.

**Results:**

A total of 5049 live births with corresponding maternal SARS-CoV-2 infection in pregnancy (mean [SD] maternal age, 31.0 [4.9] years) were matched 1:4 to 20 196 live births without maternal infection (mean [SD] maternal age, 31.1 [4.7] years). Compared with patients without infection, those with infection were more likely to be immigrants and to have high levels of material deprivation and were less likely to receive COVID-19 vaccination and live in rural areas. The crude incidence rate of any congenital anomaly was 32.5 anomalies per 1000 live births (95% CI, 27.9-37.9 anomalies per 1000 live births) with maternal SARS-CoV-2 infection and 31.1 anomalies per 1000 live births (95% CI, 28.8-33.6 anomalies per 1000 live births) without maternal SARS-CoV-2 infection (unadjusted rate ratio, 1.04; 95% CI, 0.87-1.24; *P* = .65). Multivariable logistic regression adjusting for maternal socioeconomic variables and prepregnancy COVID-19 vaccination did not alter these findings. Infection separately by trimester was not statistically significantly associated with the outcome. There were no statistically significant associations between maternal SARS-CoV-2 infection and cardiac anomalies in pregnancy overall or by trimester.

**Conclusions and Relevance:**

In this Ontario population-based study of 5049 live births with maternal SARS-CoV-2 infection matched to 20 196 live births without maternal infection, there was no association between laboratory-confirmed maternal SARS-CoV-2 infection and neonatal congenital anomalies in pregnancy overall, or by trimester of infection. These findings may provide reassurance to pregnant patients and their health care professionals, although further studies evaluating first trimester infection and risks of specific anomalies are warranted.

## Key Points

**Question:**

Is confirmed maternal SARS-CoV-2 infection during pregnancy associated with increased risk of neonatal congenital anomalies?

**Findings:**

In this population-based matched cohort study of 5049 live births with corresponding maternal SARS-CoV-2 infection in pregnancy matched to 20 196 live births without maternal infection, there was no association between maternal SARS-CoV-2 infection and neonatal congenital anomalies overall or by specific trimester of exposure.

**Meaning:**

These findings suggest that SARS-CoV-2 infection during pregnancy is unlikely to increase the risk of congenital anomalies.

## Teaser

This cohort study evaluates the associations between laboratory-confirmed maternal SARS-CoV-2 infection during pregnancy and neonatal congenital anomalies, overall during pregnancy and by specific trimester of exposure.

## Introduction

Since the onset of the COVID-19 pandemic in March 2020, substantial research has focused on maternal and perinatal outcomes. Our group previously demonstrated no effects on rates of preterm birth, stillbirth, neonatal death,^1^ preeclampsia, and severe maternal morbidity in Ontario, Canada.^2^ More recently, questions have arisen about associations between maternal SARS-CoV-2 infection in pregnancy and neonatal congenital anomalies. Congenital anomalies affect approximately 3% of births,^3,4^ with infections responsible for a small but important proportion of birth defects.^5^ The potential teratogenic effects of COVID-19 disease remain unknown. Some initial studies raised concern over associations between COVID-19 and congenital anomalies,^6^ cardiac anomalies in particular,^7,8,9^ but other studies have not corroborated these findings.^10,11^ Positive studies to date did not examine the trimester of maternal infection with SARS-CoV-2^6^ or measure the effects of the COVID-19 pandemic rather than individual SARS-CoV-2 infection.^7,9^ First trimester in utero exposure is largely responsible for other examples of congenital anomalies secondary to maternal infections, underscoring the importance of exposure timing in the determination of causality.^12^ The proposed mechanisms through which SARS-CoV-2 could potentiate teratogenic effects are hypothesized to include placental dysfunction, maternal immune activation, and direct vertical viral transmission to the fetus.^13^ Transplacental transmission has been demonstrated, showing that transmembrane serine protease 2 facilitates viral entry through angiotensin-converting enzyme 2 receptors in trophoblast cells.^14^ Confirmed neonatal cases of vertically transmitted SARS-CoV-2 infection have been reported.^15^

Further research on confirmed maternal SARS-CoV-2 infection at critical time points in gestation is required to elucidate whether teratogenic effects exist for this pathogen. The aim of this study was to evaluate associations between laboratory-confirmed SARS-CoV-2 maternal infection in pregnancy and neonatal congenital anomalies during the COVID-19 pandemic in Ontario, Canada. Secondary objectives were to separately assess associations with maternal SARS-CoV-2 infection in the first, second, and third trimesters, and to evaluate for potential confounding by maternal socioeconomic and health-related characteristics.

## Methods

### Design, Setting, and Population

This was a population-based matched cohort study of live births in Ontario, Canada, from December 14, 2020, to December 31, 2021. Health and administrative datasets used for the study were linked with unique encoded identifiers and analyzed at ICES (eTable 1 in Supplement 1). ICES is an independent, nonprofit research institute whose legal status under Ontario’s health information privacy law allows it to collect and analyze health care and demographic data, without consent, for health system evaluation and improvement. The use of the data in this project is authorized under section 45 of Ontario’s Personal Health Information Protection Act and does not require review by a research ethics board. Datasets held by ICES have previously been validated for perinatal research.^16^ We followed the Reporting of studies Conducted Using Observational Routinely-Collected Health Data (RECORD) reporting guideline, a population data extension to the Strengthening the Reporting of Observational Studies in Epidemiology (STROBE) reporting guideline.^17^

We identified all in-hospital live births at 22 weeks’ or more gestational age using the MOMBABY dataset derived from the Canadian Institute for Health Information Discharge Abstract Database. We included births with a corresponding maternal SARS-CoV-2 infection during pregnancy, confirmed by real-time polymerase chain reaction (RT-PCR) testing. The results from every RT-PCR test performed in Ontario were recorded in the COVID-19 Integrated Testing Dataset until the study end date of December 31, 2021. This date marked the end of routine SARS-CoV-2 testing and mandatory result reporting in the province. Live births with a corresponding positive maternal SARS-CoV-2 RT-PCR test in pregnancy were matched to 4 live births with no corresponding positive test identified at any time during pregnancy. The no infection group included those with a negative RT-PCR test result and those with no test performed during pregnancy. The inclusion time frame meant that all pregnancies were conceived during the COVID-19 pandemic, which was initially declared in Ontario in March 2020. This time frame meant it was unlikely that more than 1 pregnancy for the same individual was included in the cohort. We excluded stillbirths and live births that could not be linked to maternal records, those without continuous maternal or neonatal publicly funded provincial health insurance (Ontario Health Insurance Plan), and those with missing or conflicting data for gestational age at birth, neonatal sex, or date of maternal COVID-19 vaccination.

### Exposure and Matching Variables

The primary exposure was maternal SARS-CoV-2 infection during pregnancy, defined as a positive RT-PCR test between conception and birth. Estimated date of conception was calculated retrospectively from the date of birth and gestational age at birth. Births with a corresponding maternal SARS-CoV-2 infection during pregnancy were hard-matched with an exact ratio of 1:4 to births without SARS-CoV-2 infection during pregnancy. Matching was performed on maternal age (within 2 years), delivery date (within 2 weeks), gestational age at birth (within 2 weeks), neonatal sex, and prepregnancy diabetes, as this condition is a known risk factor for congenital anomalies.^18^ Matching was conducted without replacement, ensuring that a birth could not be included more than once in the cohort. We evaluated SARS-CoV-2 infection separately by trimester as secondary exposures of interest, with the first trimester defined as conception through 14 weeks of gestation, the second trimester as 15 to 28 weeks, and the third trimester as 29 weeks onward.

### Outcome and Covariates

The primary outcome was any neonatal congenital anomaly diagnosed using *International Statistical Classification of Diseases and Related Health Problems, Tenth Revision* and Canadian Classification of Interventions codes abstracted from neonatal medical records using the MOMBABY and Better Outcomes Network & Registry perinatal datasets (eTable 2 in Supplement 1).^19^ These datasets have been used previously to evaluate the relationship between prenatal exposures and neonatal congenital anomalies in Ontario.^20,21,22,23^ We evaluated congenital cardiac anomalies (critical and noncritical heart defects) as a secondary outcome. We excluded matched sets if any within the set had a congenital anomaly corresponding to a diagnosis of chromosomal abnormality or congenital TORCH (toxoplasmosis, syphilis, rubella, cytomegalovirus, or herpes simplex) infection, as these are known causes of congenital anomalies.

Maternal and pregnancy characteristics included parity (primiparous vs multiparous), multiple gestation (singleton vs twins or multiples), prepregnancy body mass index (calculated as weight in kilograms divided by height in meters squared; continuous), substance use disorder, and prenatal exposure to smoking, alcohol, cannabis, or other recreational drugs. COVID-19 vaccination before pregnancy was assessed as a binary variable (none vs any). We included information on maternal immigration status (nonimmigrant vs immigrant), rurality (urban vs rural residence), and material resource quintile, as these socioeconomic characteristics are associated with congenital cardiac anomalies in Ontario.^24,25^ Material resource quintile was obtained from the Ontario Marginalization Index and is a dimension closely connected to poverty and socioeconomic position.^26^ This index is operationalized at the neighborhood level (Dissemination Areas) by indicators derived from the Canadian Census.

### Statistical Analysis

We evaluated distributions of maternal and neonatal characteristics across groups using standardized differences, considering a standardized difference greater than or equal to 0.1 as statistically significant. We calculated crude incidence rates of congenital anomalies per 1000 live births by maternal SARS-CoV-2 infection status with 95% CI based on a Poisson distribution for the study sample overall, and by each trimester of exposure. To adjust for potential effects of unmatched covariates that were imbalanced between groups (standardized difference ≥0.1), we estimated odds ratios (ORs) and 95% CIs for the association between SARS-CoV-2 infection at any time in pregnancy and congenital anomalies with multivariable conditional logistic regression. Statistical significance was set a priori at an α level of .05. Analyses were conducted between May and August 2025 using SAS statistical software version 9.4 (SAS Institute).

## Results

There were 145 188 births during the study time frame in Ontario, of which 132 773 (91.4%) met eligibility criteria. A total of 5176 live births (3.9%) had corresponding maternal SARS-CoV-2 infection in pregnancy based on a positive maternal RT-PCR test. Of these, 5073 (98.0%) were successfully matched 1:4 to live births without maternal SARS-CoV-2 infection in pregnancy. Following matching, 24 match sets (24 infection and 96 no infection) were excluded for a diagnosis of chromosomal abnormality or congenital TORCH infection, resulting in a final sample of 5049 live births with maternal SARS-CoV-2 infection matched to 20 196 live births without maternal infection (Figure).

**Figure.**
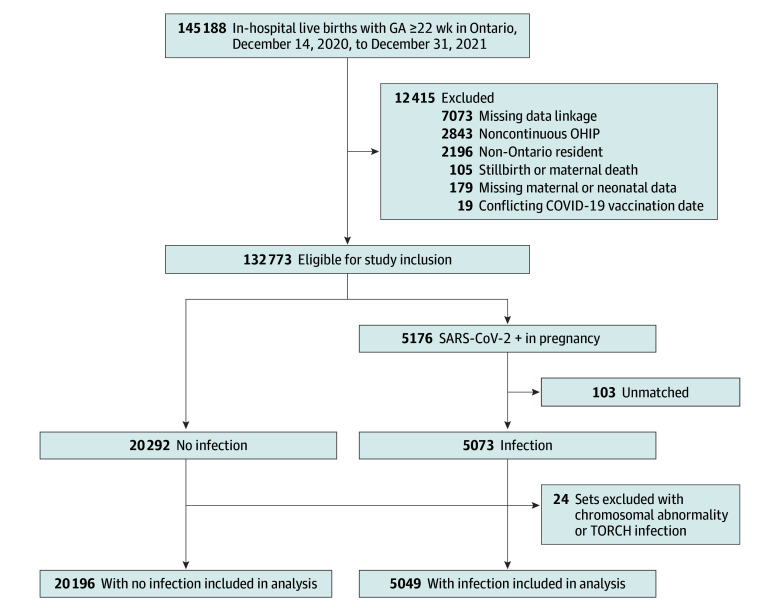
Flow Diagram of Study Inclusion and Exclusion Criteria GA indicates gestational age; OHIP, Ontario Health Insurance Plan; and TORCH, toxoplasmosis, syphilis, rubella, cytomegalovirus, herpes simplex.

Table 1 shows baseline maternal and neonatal characteristics. The distributions of matched variables were similar between groups. The mean (SD) maternal age was 31.0 (4.9) years for the SARS-CoV-2 infection group and 31.1 (4.7) years for the no infection group. Prepregnancy diabetes occurred in 2.3% of both groups (472 participants in the no infection group vs 118 participants in the infection group). The mean (SD) gestational age at birth was 38.7 (1.6) weeks for infection and 38.5 (1.7) weeks for no infection groups (mean difference, 1.3 days). The median (IQR) number of days between birth dates for match sets was 0 (0-0) days, and 95% of sets were matched within 1 day (eFigure in Supplement 1). For the unmatched covariates, more than one-half of pregnant patients were multiparous in both groups (2951 patients [58.4%] in the infection group and 11 087 patients [54.9%] in the no infection group), with similar rates of twins or multiples (130 patients [2.6%] in the infection group vs 460 patients [2.3%] in the no infection group). Prepregnancy COVID-19 vaccination differed, with 11 602 patients (57.4%) vaccinated in the no SARS-CoV-2 infection group and 2419 patients (47.9%) vaccinated in the infection group (standardized difference, 0.19). The distribution across maternal material resource quintiles also differed. Those in the infection group were more likely to live in areas with the highest material deprivation (quintile 5, 1240 patients [24.6%]) compared with the no infection group (3940 patients [19.5%]; standardized difference, 0.12). Patients with SARS-CoV-2 infection were more likely to have immigrated to Canada (2309 patients [45.7%] vs 5370 patients [26.6%]; standardized difference, 0.41) and less likely to live in rural areas (259 patients [5.1%] vs 2612 patients [12.9%]; standardized difference, 0.28). Within the infection group, the distribution of SARS-CoV-2 infection was similar across trimesters, with 1492 infections (29.6%) occurring in the first trimester, 1796 (35.6%) in the second trimester, and 1761 (34.9%) in the third trimester.

**Table 1.** Baseline Maternal and Neonatal Characteristics for the Matched Study Sample

Characteristic	Participants, No. (%) (N = 25 245)		Standardized difference
	No SARS-CoV-2 (n = 20 196)	SARS-CoV-2 (n = 5049)	
Maternal age, y^a^			
Mean (SD)	31.1 (4.7)	31.0 (4.9)	0.015
Median (IQR)	31 (28-34)	31 (28-34)	0.018
Multiparous	11 087 (54.9)	2951 (58.4)	0.072
Maternal body mass index^b^			
Mean (SD)	26.2 (6.7)	26.3 (6.6)	0.018
Median (IQR)	25 (22-29)	25 (22-29)	0.041
Missing, %	83.6	85.8	NA
Prepregnancy diabetes^a^	472 (2.3)	118 (2.3)	0.000
Drug or substance use			
No	3584 (17.7)	880 (17.4)	0.008
Yes	30 (0.1)	8 (0.2)	0.003
Missing	16 582 (82.1)	4161 (82.4)	0.008
Alcohol use			
No	3551 (17.6)	892 (17.7)	0.002
Yes	85 (0.4)	11 (0.2)	0.036
Missing	16 568 (82.0)	4148 (82.1)	0.003
Smoking			
No	3545 (17.6)	891 (17.6)	0.002
Yes	88 (0.4)	11 (0.2)	0.038
Missing	16 563 (82.0)	4147 (82.1)	0.003
Cannabis use			
No	3411 (16.9)	827 (16.4)	0.014
Yes	159 (0.8)	20 (0.4)	0.051
Missing	16 626 (82.3)	4202 (83.2)	0.024
COVID-19 vaccination	11 602 (57.4)	2419 (47.9)	0.19
Immigrant	5370 (26.6)	2309 (45.7)	0.41
Rural residence	2612 (12.9)	259 (5.1)	0.275
Material resource quintile			
1 (Lowest deprivation)	4083 (20.2)	626 (12.4)	0.21
2	4490 (22.2)	983 (19.5)	0.068
3	3955 (19.6)	1073 (21.3)	0.041
4	3590 (17.8)	1091 (21.6)	0.096
5 (Highest deprivation)	3940 (19.5)	1240 (24.6)	0.12
Missing	138 (0.7)	36 (0.7)	0.004
Gestational age at birth, wk^a^			
Mean (SD)	38.7 (1.6)	38.5 (1.7)	0.11
Median (IQR)	39 (38-40)	39 (38-40)	0.11
Neonatal sex^a^			
Female	10 028 (49.7)	2507 (49.7)	0.000
Male	10 168 (50.3)	2542 (50.3)	0.000
Multiplicity			
Singleton	19 736 (97.7)	4919 (97.4)	0.019
Twins or multiples	460 (2.3)	130 (2.6)	0.019

Abbreviation: NA, not applicable.

^a^ Denotes variables included in match.

^b^ Body mass index is calculated as weight in kilograms divided by height in meters squared.

Congenital anomalies were present in 164 neonates (3.2%) in the SARS-CoV-2 infection group and 628 neonates (3.1%) in the no infection group. Noncritical cardiac defects were the most common congenital anomaly (52 neonates [1.0%] in the infection group vs 176 neonates [0.9%] in the no infection group; standardized difference, 0.016) (eTable 3 in Supplement 1). The crude incidence rate of any congenital anomaly was 32.5 per 1000 live births (95% CI, 27.9-37.9 per 1000 live births) in the SARS-CoV-2 infection group and 31.1 per 1000 live births (95% CI, 28.8-33.6 per 1000 live births) in the no infection group (Table 2). The rate ratio (RR) for SARS-CoV-2 infection at any time in pregnancy was not statistically significant (RR, 1.04; 95% CI, 0.87-1.24; *P* = .65). The crude incidence rate of congenital anomalies associated with first trimester infection was 34.9 per 1000 live births (95% CI, 26.6-45.7 per 1000 live births) compared with 31.8 per 1000 live births (95% CI, 27.6-36.7 per 1000 live births) in the no infection group (RR, 1.09; 95% CI, 0.79-1.49; *P* = .61). Incidence rates did not differ statistically significantly between groups in the second or third trimesters.

**Table 2.** Crude IRs and RRs of Neonatal Congenital Anomalies by Maternal SARS-CoV-2 Infection in Pregnancy

Maternal SARS-CoV-2 infection	No. at risk	No. of cases	IR/1000 live births (95% CI)	RR (95% CI)	*P* value
Any infection					
Yes	5049	164	32.5 (27.9-37.9)	1.04 (0.87-1.24)	.65
No	20 196	628	31.1 (28.8-33.6)	1 [Reference]	
Infection by trimester					
First trimester (conception to 14 wk)					
Yes	1492	52	34.9 (26.6-45.7)	1.09 (0.79-1.49)	.61
No	5968	190	31.8 (27.6-36.7)	1 [Reference]	
Second trimester (15-28 wk)					
Yes	1796	60	33.4 (25.9-43.0)	1.07 (0.79-1.43)	.68
No	7184	224	31.2 (27.4-35.5)	1 [Reference]	
Third trimester (≥29 wk)					
Yes	1761	52	29.5 (22.5-38.8)	0.97 (0.70-1.32)	.93
No	7044	214	30.4 (26.6-34.7)	1 [Reference]	

Abbreviations: IR, incidence rate; RR, rate ratio.

Cardiac anomalies were present in 54 neonates (1.1%) in the SARS-CoV-2 infection group and 177 (0.9%) in the no infection group (Table 3). The crude incidence rate was 10.7 per 1000 live births (95% CI, 8.2-14.0 per 1000 live births) in the infection group and 8.8 per 1000 live births (95% CI, 7.6-10.2 per 1000 live births) in the no infection group, which was not statistically significant (RR, 1.22; 95% CI, 0.88-1.66 per 1000 live births; *P* = .23). The crude incidence of cardiac anomalies was higher for first trimester infection compared with no exposure (14.1 per 1000 live births [95% CI, 9.2-21.6 per 1000 live births] in the infection group vs 8.0 per 1000 live births [95% CI, 6.1-10.7 per 1000 live births] in the no infection group) but the difference was not statistically significant (RR, 1.75; 95% CI, 1.00-2.98; *P* = .05). There were no statistically significant associations between second or third trimester infection and cardiac anomalies.

**Table 3.** Crude IRs and RRs of Neonatal Cardiac Anomalies by Maternal SARS-CoV-2 Infection in Pregnancy

Maternal SARS-CoV-2 infection	No. at risk	No. of cases	IR/1000 live births (95% CI)	RR (95% CI)	*P* value
Any infection					
Yes	5049	54	10.7 (8.2-14.0)	1.22 (0.88-1.66)	.23
No	20 196	177	8.8 (7.6-10.2)	1 [Reference]	
Infection by trimester					
First trimester (conception to 14 wk)					
Yes	1492	21	14.1 (9.2-21.6)	1.75 (1.00-2.98)	.05
No	5968	48	8.0 (6.1-10.7)	1 [Reference]	
Second trimester (15-28 wk)					
Yes	1796	18	10.0 (6.3-15.9)	0.92 (0.52-1.56)	.88
No	7184	78	10.9 (8.7-13.6)	1 [Reference]	
Third trimester (≥29 wk)					
Yes	1761	15	8.5 (5.1-14.1)	1.18 (0.61-2.13)	.67
No	7044	51	7.2 (5.5-9.5)	1 [Reference]	

Abbreviations: IR, incidence rate; RR, rate ratio.

The unadjusted OR of maternal SARS-CoV-2 infection at any time in pregnancy was not statistically significantly associated with congenital anomalies (OR, 1.05; 95% CI, 0.88-1.25) (Table 4). Adjusting for immigration status, rurality, material resource quintile, and prepregnancy COVID-19 vaccination made no material difference (OR, 1.03; 95% CI, 0.86-1.23). For the secondary outcome of cardiac anomalies, there was no association following adjustment for these variables (OR, 1.23; 95% CI, 0.88-1.71).

**Table 4.** Multivariable Logistic Regression Models for Maternal SARS-CoV-2 Infection in Pregnancy and Congenital Anomaly Outcomes

Outcome	OR (95% CI)	
	Unadjusted	Adjusted^a^
Congenital anomaly (any)	1.05 (0.88-1.25)	1.03 (0.86-1.23)
Cardiac anomaly	1.25 (0.90-1.71)	1.23 (0.88-1.71)

Abbreviation: OR, odds ratio.

^a^ Adjusted for immigration status, rurality, material resource quintile, and prepregnancy COVID-19 vaccination.

## Discussion

This population-based matched cohort study found no evidence of an association between maternal SARS-Cov-2 infection in pregnancy and neonatal congenital anomalies. We assessed these associations by trimester of infection and found no significant associations with the outcome. We found no evidence of an association with cardiac anomalies as a secondary outcome, although the crude incidence estimates were higher for first trimester SARS-CoV-2 infection. Noncritical cardiac defects represented the largest category of congenital anomalies in this study; however, these numbers were low overall in both groups. Following multivariable adjustment, there remained no association between maternal SARS-CoV-2 infection and neonatal cardiac anomalies.

To our knowledge, this is the largest population-based study to date using confirmatory RT-PCR testing to define SARS-CoV-2 infection with information on infection timing during pregnancy. Some prior cohort studies found higher rates of congenital anomalies associated with maternal SARS-CoV-2 infection or with the COVID-19 pandemic era, with a particular signal suggested for cardiac anomalies.^6,7,8,9^ An analysis of perinatal outcomes using data from a health maintenance organization in Southern California demonstrated elevated odds of any congenital anomaly associated with confirmed maternal SARS-CoV-2 infection (adjusted OR, 1.69; 95% CI, 1.15-2.50).^6^ However, that study performed routine testing at maternal admission for birth, and over 70% of infections were identified in the third trimester. This infection timing is unlikely to substantiate a biologically plausible link with congenital anomalies, which would be expected to occur with exposures at earlier gestation during embryonic development. Furthermore, the study included both chromosomal and nonchromosomal congenital anomalies in the outcome, and the former would not conceivably be related to maternal SARS-CoV-2 infection.

In contrast, our findings align with existing population-based cohort studies from Scotland^10^ and Quebec,^11^ which did not find associations with congenital anomalies. Those studies were smaller than the present study, the largest having only 9 cases with confirmed first trimester maternal SARS-CoV-2 infection.^11^ In that study, Auger et al^11^ did find an association between congenital anomalies identifiable by physical examination and the COVID-19 pandemic era, but no association with maternal SARS-CoV-2 infection, suggesting a surveillance effect of the pandemic itself on the diagnosis of these conditions. This highlights an important distinction in the assessment of evidence for SARS-CoV-2 teratogenic effects, as only a handful of studies to date assessed confirmed maternal infection rather than pandemic era effects. Research focused on cardiac anomalies has found associations with the COVID-19 pandemic, including situs inversus identified by antenatal ultrasound^7^ and cyanotic cardiac lesions identified using US birth certificate data.^9^ Both studies used historical controls and evaluated pandemic effects rather than the effect of individual, confirmed SARS-CoV-2 infection. The latter study by Khalil et al^9^ raises important questions around antenatal diagnosis and pregnancy care access, which might have conceivably differed between time periods before and during the COVID pandemic. However, again, the evidence for a plausible link between COVID disease and these outcomes is not strongly supported by their analysis given the lack of individual-level data on SARS-CoV-2 infection during pregnancy.

Our study expands the literature with a substantially larger sample size. SARS-CoV-2 infection in pregnancy was confirmed by positive RT-PCR test, and analyses were performed for pregnancy overall and separately by trimester. Infection timing was evenly divided among the 3 trimesters, allowing for more comprehensive analysis of trimester-specific risks compared with previous cohort data. We used Ontario administrative and health datasets held by ICES that reliably capture all in-hospital births (98% of births in Ontario) and are validated for use in perinatal research.^16^ We were able to identify and exclude cases associated with chromosomal abnormalities and congenital TORCH infections. We accounted for known risk factors associated with congenital anomalies by matching on prepregnancy diabetes.^18^ We adjusted for rurality, material resource deprivation, and immigration status as these social determinants of health differed between groups and are associated with congenital anomalies in Ontario.^24,25^ These datasets have been previously used to evaluate associations between prenatal exposures and neonatal outcomes in the province.^20,21,22,23^

### Limitations

This study had some limitations. SARS-CoV-2 infection was assessed according to a positive RT-PCR test in pregnancy. The matches had either a negative RT-PCR test or no test performed in pregnancy. Although all laboratory-based SARS-CoV-2 testing was provincially reported during the study period, it is possible that patients with infection did not present for testing and would be misclassified as having no infection. Results from home-based SARS-CoV-2 tests were not reported; however, home testing was not widely available during the study period. Fetal in utero infection could not be assessed in this study. We matched on gestational age at birth, and it is possible that this could have obscured an association between SARS-CoV-2 infection and congenital anomalies that are associated with gestational age at birth. Maternal obesity is a risk factor for congenital anomalies,^27,28^ and although the groups did not differ with respect to body mass index, there was a high degree of missing data for this variable. Similarly, substance use variables had high degrees of missingness. There is varying evidence for each of these as risk factors for congenital anomalies.^29,30,31^ In our study, there did not appear to be differences in the proportion of patients using substances between groups. We did not include stillbirths or pregnancy terminations in the study as the cause or indication could not be identified in the datasets. We examined the number of stillbirths occurring at 20 weeks of gestation or later in the MOMBABY cohort and found these numbers were too small for meaningful comparison. This could have introduced bias if a higher proportion of stillbirths occurred in the SARS-CoV-2 infection group, particularly if the cause of stillbirth was associated with congenital anomaly. Our previous work demonstrated that rates of stillbirth in Ontario were not associated with the COVID-19 pandemic, although that study did not assess maternal SARS-CoV-2 infection directly.^1^ We did not assess specific SARS-CoV-2 variants or infection severity, which represent areas for future research. In addition, although we found no evidence of an association between maternal SARS-CoV-2 infection and cardiac anomalies, the crude incidence rate for first trimester infection was higher than that for the group without infections. We hesitate to elaborate on this finding given the small numbers in both groups; however, this warrants further research.

## Conclusions

In this Ontario population-based study of 5049 live births with maternal SARS-CoV-2 infection matched to 20 196 live births without maternal infection, we found no association between laboratory-confirmed maternal SARS-CoV-2 infection and neonatal congenital anomalies in pregnancy overall or by trimester of infection. These findings may provide reassurance to pregnant patients and their health care professionals, although further studies evaluating first trimester infection and risks of specific anomalies are warranted.

## Notes

Supplement 1. eFigure. Index date distribution for SARS-CoV-2 infection (exposed) and no infection (unexposed) match sets eTable 1. Datasets used in study eTable 2. *ICD-10* and CCI codes for fetal/neonatal congenital anomalies eTable 3. Distribution of congenital anomalies among SARS-CoV-2 infection groups

Supplement 2. Data Sharing Statement

## References

[1] Simpson AN, Snelgrove JW, Sutradhar R, Everett K, Liu N, Baxter NN. Perinatal outcomes during the COVID-19 pandemic in Ontario, Canada. JAMA Netw Open. 2021;4(5):e2110104. doi:10.1001/jamanetworkopen.2021.10104 33978727 PMC8116980

[2] Snelgrove JW, Simpson AN, Sutradhar R, Everett K, Liu N, Baxter NN. Preeclampsia and severe maternal morbidity during the COVID-19 pandemic: a population-based cohort study in Ontario, Canada. J Obstet Gynaecol Can. 2022;44(7):777-784. doi:10.1016/j.jogc.2022.03.008 35395419 PMC8979839

[3] Boyle B, Addor MC, Arriola L, . Estimating global burden of disease due to congenital anomaly: an analysis of European data. Arch Dis Child Fetal Neonatal Ed. 2018;103(1):F22-F28. doi:10.1136/archdischild-2016-311845 28667189 PMC5750368

[4] Stallings EB, Isenburg JL, Rutkowski RE, ; National Birth Defects Prevention Network. National population-based estimates for major birth defects, 2016-2020. Birth Defects Res. 2024;116(1):e2301. doi:10.1002/bdr2.2301 38277408 PMC10898112

[5] Ssentongo P, Hehnly C, Birungi P, . Congenital cytomegalovirus infection burden and epidemiologic risk factors in countries with universal screening: a systematic review and meta-analysis. JAMA Netw Open. 2021;4(8):e2120736. doi:10.1001/jamanetworkopen.2021.20736 34424308 PMC8383138

[6] Getahun D, Peltier MR, Lurvey LD, . Association between SARS-CoV-2 infection and adverse perinatal outcomes in a large health maintenance organization. Am J Perinatol. 2024;41(2):199-207. doi:10.1055/s-0042-1749666 35738286

[7] Wang Y, Guo Z, Ye B, . Association of SARS-CoV-2 infection during early weeks of gestation with situs inversus. N Engl J Med. 2023;389(18):1722-1724. doi:10.1056/NEJMc2309215 37913512 PMC10755830

[8] Li Y, Wang Y, Wu H, . Increased risk of fetal left-right asymmetry disorders associated with maternal SARS-CoV-2 infection during the first trimester. Sci Rep. 2024;14(1):11422. doi:10.1038/s41598-024-61778-w 38763951 PMC11102919

[9] Khalil A, Painter I, Souter V. Congenital heart defects during COVID-19 pandemic. Ultrasound Obstet Gynecol. 2025;65(5):546-551. doi:10.1002/uog.29126 39541959

[10] Calvert C, Carruthers J, Denny C, . A population-based matched cohort study of major congenital anomalies following COVID-19 vaccination and SARS-CoV-2 infection. Nat Commun. 2023;14(1):107. doi:10.1038/s41467-022-35771-8 36609574 PMC9821346

[11] Auger N, Arbour L, Lewin A, Brousseau É, Healy-Profitós J, Luu TM. Congenital anomalies during Covid-19: artifact of surveillance or a real TORCH? Eur J Epidemiol. 2024;39(6):613-621. doi:10.1007/s10654-024-01122-8 38589643

[12] Rasmussen SA, Jamieson DJ, Honein MA, Petersen LR. Zika virus and birth defects—reviewing the evidence for causality. N Engl J Med. 2016;374(20):1981-1987. doi:10.1056/NEJMsr1604338 27074377

[13] Stolojanu C, Doros G, Bratu ML, . COVID-19 and its potential impact on children born to mothers infected during pregnancy: a comprehensive review. Diagnostics (Basel). 2024;14(21):2443. doi:10.3390/diagnostics14212443 39518410 PMC11545714

[14] Ashary N, Bhide A, Chakraborty P, . Single-cell RNA-seq identifies cell subsets in human placenta that highly expresses factors driving pathogenesis of SARS-CoV-2. Front Cell Dev Biol. 2020;8:783. doi:10.3389/fcell.2020.00783 32974340 PMC7466449

[15] Jeganathan K, Paul AB. Vertical transmission of SARS-CoV-2: a systematic review. Obstet Med. 2022;15(2):91-98. doi:10.1177/1753495X211038157 35795545 PMC9247633

[16] Joseph KS, Fahey J; Canadian Perinatal Surveillance System. Validation of perinatal data in the Discharge Abstract Database of the Canadian Institute for Health Information. Chronic Dis Can. 2009;29(3):96-100. doi:10.24095/hpcdp.29.3.01 19527567

[17] Benchimol EI, Smeeth L, Guttmann A, ; RECORD Working Committee. The REporting of studies Conducted using Observational Routinely-collected health Data (RECORD) statement. PLoS Med. 2015;12(10):e1001885. doi:10.1371/journal.pmed.1001885 26440803 PMC4595218

[18] Liu S, Rouleau J, León JA, Sauve R, Joseph KS, Ray JG; Canadian Perinatal Surveillance System. Impact of pre-pregnancy diabetes mellitus on congenital anomalies, Canada, 2002-2012. Health Promot Chronic Dis Prev Can. 2015;35(5):79-84. doi:10.24095/hpcdp.35.5.01 26186019 PMC4910455

[19] World Health Organization. International Statistical Classification of Diseases, Tenth Revision (ICD-10). World Health Organization; 1992.

[20] Simard C, Fu L, Odugbemi T, . Exposure to computed tomography before pregnancy and risk for pregnancy loss and congenital anomalies: a population-based cohort study. Ann Intern Med. 2025;178(11):1539-1548. doi:10.7326/ANNALS-24-03479 40921077

[21] Sattolo ML, Arbour L, Bilodeau-Bertrand M, Lee GE, Nelson C, Auger N. Association of birth defects with child mortality before age 14 years. JAMA Netw Open. 2022;5(4):e226739. doi:10.1001/jamanetworkopen.2022.6739 35404459 PMC9002336

[22] Miao Q, Dunn S, Wen SW, . Association between maternal marginalization and infants born with congenital heart disease in Ontario Canada. BMC Public Health. 2023;23(1):790. doi:10.1186/s12889-023-15660-5 37118769 PMC10142402

[23] Jorgensen SCJ, Drover SSM, Fell DB, ; Canadian Immunization Research Network (CIRN) Provincial Collaborative Network (PCN) Investigators. Association between maternal mRNA covid-19 vaccination in early pregnancy and major congenital anomalies in offspring: population based cohort study with sibling matched analysis. BMJ Med. 2024;3(1):e000743. doi:10.1136/bmjmed-2023-000743 39574424 PMC11579536

[24] Miao Q, Dunn S, Wen SW, Lougheed J, Sharif F, Walker M. Associations of congenital heart disease with deprivation index by rural-urban maternal residence: a population-based retrospective cohort study in Ontario, Canada. BMC Pediatr. 2022;22(1):476. doi:10.1186/s12887-022-03498-6 35931992 PMC9356510

[25] Miao Q, Dunn S, Wen SW, . Neighbourhood maternal socioeconomic status indicators and risk of congenital heart disease. BMC Pregnancy Childbirth. 2021;21(1):72. doi:10.1186/s12884-020-03512-8 33478420 PMC7819193

[26] Matheson FI, Moloney G, van Ingen T. 2021 Ontario marginalization index: user guide. St. Michael’s Hospital, Unit Health Toronto. July 2023. Accessed March 27, 2026. https://www.publichealthontario.ca/-/media/documents/o/2017/on-marg-userguide.pdf

[27] Rankin J, Tennant PW, Stothard KJ, Bythell M, Summerbell CD, Bell R. Maternal body mass index and congenital anomaly risk: a cohort study. Int J Obes (Lond). 2010;34(9):1371-1380. doi:10.1038/ijo.2010.66 20368710

[28] Iessa N, Bérard A. Update on prepregnancy maternal obesity: birth defects and childhood outcomes. J Pediatr Genet. 2015;4(2):71-83. doi:10.1055/s-0035-1556739 27617118 PMC4918711

[29] Tadesse AW, Ayano G, Dachew BA, . The association between prenatal cannabis use and congenital birth defects in offspring: a cumulative meta-analysis. Neurotoxicol Teratol. 2024;102:107340. doi:10.1016/j.ntt.2024.107340 38460861

[30] Liu S, Joseph KS, Lisonkova S, ; Canadian Perinatal Surveillance System (Public Health Agency of Canada). Association between maternal chronic conditions and congenital heart defects: a population-based cohort study. Circulation. 2013;128(6):583-589. doi:10.1161/CIRCULATIONAHA.112.001054 23812182

[31] Hackshaw A, Rodeck C, Boniface S. Maternal smoking in pregnancy and birth defects: a systematic review based on 173 687 malformed cases and 11.7 million controls. Hum Reprod Update. 2011;17(5):589-604. doi:10.1093/humupd/dmr022 21747128 PMC3156888

