# Supplementary material for: SARS-CoV-2 Infection and Rates of Neonatal Congenital Anomalies
Source: JAMA Netw Open. 2026 May 7;9(5):e2611440. doi: 10.1001/jamanetworkopen.2026.11440 (PMC13153994; doi:10.1001/jamanetworkopen.2026.11440)
Supplement: Supplement 1. — eFigure. Index date distribution for SARS-CoV-2 infection (exposed) and no infection (unexposed) match sets eTable 1. Datasets used in study eTable 2. ICD-10 and CCI codes for fetal/neonatal congenital anomalies eTable 3. Distribution of congenital anomalies among SARS-CoV-2 infection groups [file jamanetwopen-e2611440-s001.pdf]

Supplemental Online Content

Snelgrove JW, Sutradhar R, Baxter NN, et al. SARS-CoV-2 infection and rates of neonatal congenital anomalies. *JAMA Netw Open*. 2026;9(5):e2611440.
doi:10.1001/jamanetworkopen.2026.11440

eFigure. Index date distribution for SARS-CoV-2 infection (exposed) and no infection (unexposed) match sets

eTable 1. Datasets used in study

eTable 2. *ICD-10* and CCI codes for fetal/neonatal congenital anomalies

eTable 3. Distribution of congenital anomalies among SARS-CoV-2 infection groups

This supplemental material has been provided by the authors to give readers additional information about their work.

© 2026 Snelgrove JW et al. *JAMA Network Open*.

eFigure. Index date distribution for SARS-CoV-2 infection (exposed) and no infection (unexposed) match sets.

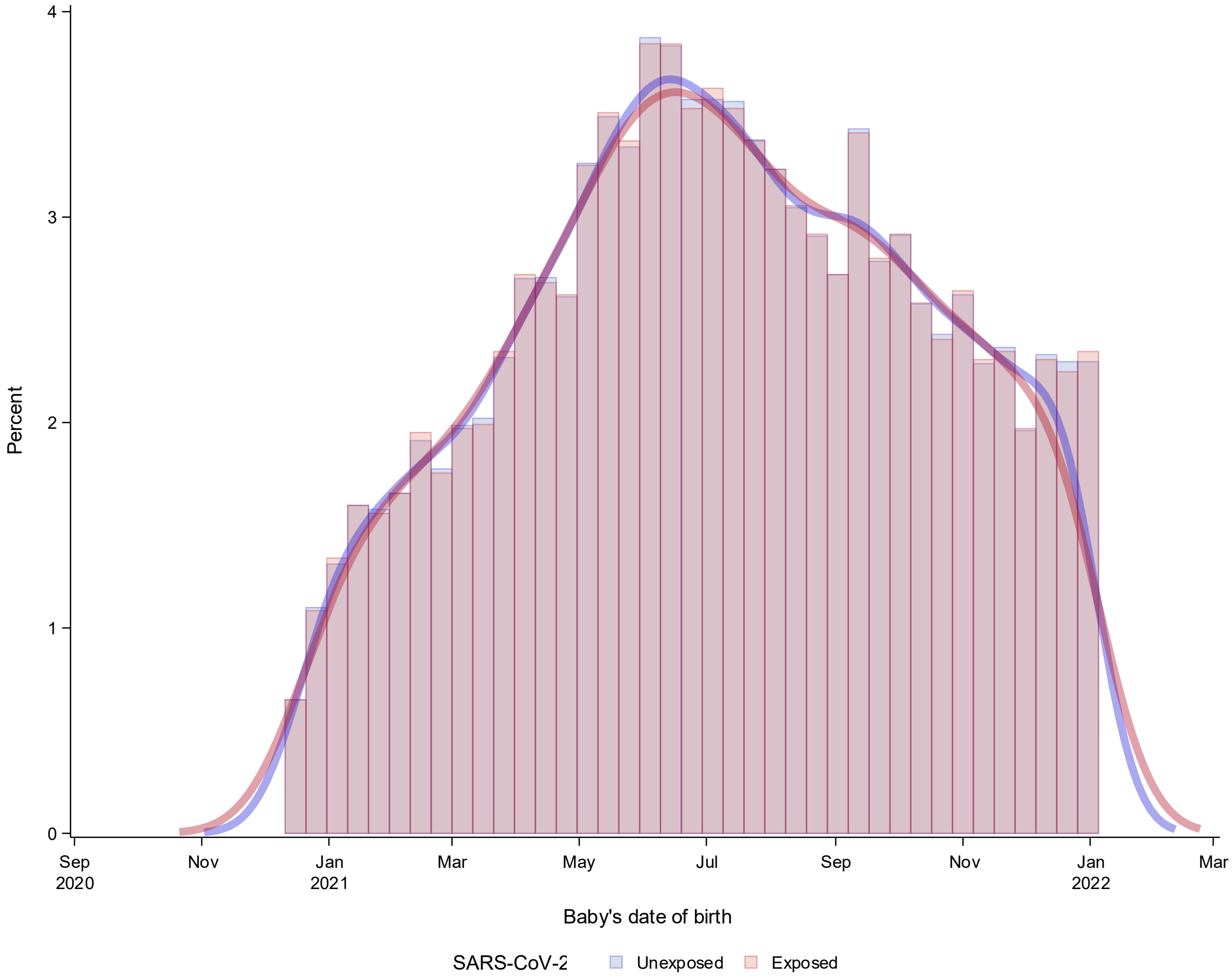

© 2026 Snelgrove JW et al. *JAMA Network Open*.

eTable 1. Datasets used in study

Database	Description	Use in Current Study
BORN: Better Outcomes Registry and Network	A perinatal dataset of pregnancy, birth, and neonatal information for all hospital-based births in Ontario.	Used to identify congenital anomaly outcomes.<br>Used to determine TORCH infection (exclusion criteria)<br>Used to define covariates: maternal body mass index (BMI), prenatal smoking, cannabis, alcohol
C19INTGR: COVID-19 Integrated Testing Data	A comprehensive dataset of all available SARS-CoV-2 diagnostic laboratory results in Ontario.	Identifies whether the test result was positive, negative, or indeterminate. Indeterminate test results were assumed to be negative.<br>Used to determine SARS-CoV-2 exposure status and gestational age on day of test.
CENSUS: Ontario Census Area Profiles	A national census of the Canadian population conducted every five years by Statistics Canada	Used to identify whether a person's residence is geographically defined as rural or urban.
CIHI-DAD: Canadian Institute for Health Information Discharge Abstract Database	Contains clinical, demographic, and administrative data on all hospital discharges in Ontario, including diagnostic coding of the most responsible diagnosis at discharge.	Used to identify congenital anomaly outcomes.<br>Used to determine aneuploidy (exclusion criteria) and substance use disorder (covariate)
COVaxON: Ontario COVID-19 Vaccine Data	Contains the records of all SARS-CoV-2 vaccination events in the province including the date, dose, reason for, product used, and location of administration	Used to identify maternal vaccination status based on receipt of a mRNA vaccine.
ICES-derived cohorts	Cumulative datasets created using validated case-finding algorithms to identify individuals with specific diseases. The algorithms typically use a combination of hospital, emergency department, and outpatient data, and in some instances drug claim information, and specify a combination of a certain number of records,	Used to identify persons with pre-pregnancy diabetes

© 2026 Snelgrove JW et al. *JAMA Network Open*.

	coupled with diagnostic codes, that must have occurred within a certain time period.	
IRCC: Immigration, Refugees and Citizenship Canada Permanent Resident Database	Contains records of permanent residents who landed in Ontario from January 1985 onward and includes landing date and demographic information.	Used to determine a person's immigrant class and recency of immigration.
MOMBABY	Contains pregnancy and birth-related information obtained during a birth hospitalization. Derived from the CIHI-DAD.	Used to identify in-hospital obstetrical births during the study period, and gestational age and parity at the time of the index test.
ONMARG: Ontario Marginalization Index	A geographically based (Census) measure that quantifies the degree of marginalization in Ontario.	Used to estimate individuals' material resource quintile.<br>Quintile 1 lowest deprivation<br>Quintile 2<br>Quintile 3<br>Quintile 4<br>Quintile 5 highest deprivation
PCCF: Postal Code Conversion File	Links the postal code associated with an individual's residence to census geography	Used to assign persons to an Ontario public health region.<br>Used to assign persons to neighbourhood-level geography for SARS-C0V-2 risk factor information including recent immigration, and lower income
RPDB: Registered Persons Database	Contains a record for all individuals who have ever been registered under Ontario's public insurance plan and includes demographic information and date of death, if applicable.	Used to determine study eligibility including a valid health card number, residence in Ontario, age at index within study parameters, non-missing birthdate, non-missing postal code, and sex recorded as female.

© 2026 Snelgrove JW et al. *JAMA Network Open*.

eTable 2. *ICD-10* and CCI codes for fetal/neonatal congenital anomalies

**Critical heart defect**	
Transposition of great vessels	Q20.1-Q20.3, Q20.50
Tetralogy of Fallot	Q21.3 / 1.LD.84, 1.HP.87
Hypoplastic left heart	Q23.4
Coarctation of aorta	Q25.1
Other critical	Q20.0, Q20.4, Q21.4, Q22.0, Q22.4, Q22.5, Q26.2 / 1.LA.84
**Noncritical heart defect**	
Ventricular septum	Q21.0, Q21.8 / 1.HR.80
Atrial septum	Q21.1 / 1.HN.80
Other noncritical	Q20.58, Q20.6-Q20.9, Q21.2, Q21.9, Q22.1-Q22.3, Q22.6, Q22.8-Q23.3, Q23.8-Q25.0, Q25.2-Q26.1, Q26.3-Q28, Q89.3 / 1.LC.84
**Central nervous system**	
Neural tube defects	Q00, Q01, Q05, Q07.0 / 1.AW.72, 1.AX.73
Microcephaly	Q02
Hydrocephalus	Q03 / 1.AC.52-1.AC.54
Other central nervous system	Q04, Q06, Q07.8-Q07.9
	Q35-Q37 / 1.FB.86, 1.YE.80
**Orofacial cleft**	
**Eye, ear, nose**	
Eye	Q10-Q15, H26 / 1.CL.59, 1.CL.89
Ear	Q16-Q17
Nose	Q30
**Respiratory**	
Lung malformation	Q33
Other respiratory	Q31, Q32, Q34
**Digestive**	
Biliary or intestinal atresia	Q39.0, Q39.1, Q41, Q42, Q44.2, Q44.3
Other digestive	K31.1, Q38.0, Q38.2-Q38.8, Q39.2-Q40, Q43-Q44.1, Q44.4-Q45 / 1.NE.72
**Abdominal wall**	
Diaphragmatic hernia	Q79.0
Omphalocele	Q79.2
Gastroschisis	Q79.3 / 1.SY.84
Other abdominal wall defect	Q79.1, Q79.5
**Urinary**	
Renal agenesis	Q60
Other urinary defect	Q61-Q63, Q64.1-Q64.9
**Genital**	
Female genital defect	Q50.0, Q50.3, Q50.4, Q50.6, Q51-Q52.2, Q52.4, Q52.6, Q52.8, Q52.9
Male genital defect	Q54, Q55, Q64.0
Indeterminate sex	Q56
**Musculoskeletal**	
Congenital hip dislocation	Q65
Clubfoot	Q66 / 1.WE.72
Polydactyly, syndactyly	Q69, Q70 / 1.UF.84, 1.UI.71, 1.UJ.71, 1.WL.71

© 2026 Snelgrove JW et al. *JAMA Network Open*.

Limbs and digits	Q71.0-Q71.5, Q71.8-Q73
Other musculoskeletal	Q68.1-Q68.8, Q71.6, Q74-Q75.2, Q75.4-Q75.9, Q76.1-Q76.3, Q76.8-Q78, Q79.4, Q79.6-Q79.9
**Other**	Q80-Q82.4, Q82.9-Q83.2, Q83.8-Q84.4, Q84.8-Q87, Q89.0-Q89.2, Q89.4-Q89.8
**Chromosomal (excluded)**	
Down syndrome	Q90
Trisomy 13 and 18	Q91
Other chromosomal	Q92-Q99

© 2026 Snelgrove JW et al. *JAMA Network Open*.

eTable 3. Distribution of congenital anomalies among SARS-CoV-2 infection groups

Congenital anomaly	No SARS-CoV2 (n=20,196), No. (%)	SARS-CoV2 (n=5,049), No. (%)	SD
All	628 (3.1%)	164 (3.2%)	0.008
Critical heart defect	12 (0.1%)	7 (0.1%)	0.025
Non-critical heart defect	176 (0.9%)	52 (1.0%)	0.016
CNS	53 (0.3%)	19 (0.4%)	0.020
Orofacial	44 (0.2%)	9 (0.2%)	0.009
Respiratory	16 (0.1%)	9 (0.2%)	0.028
Digestive	53 (0.3%)	14 (0.3%)	<0.01
Abdominal	17 (0.1%)	1-5 [a]	<0.01
Urinary	134 (0.7%)	42 (0.8%)	0.020
Genital	57 (0.3%)	15 (0.3%)	<0.01
Musculoskeletal	139 (0.7%)	26 (0.5%)	0.022
Other	18 (0.1%)	12 (0.2%)	0.037

[a]small cell numbers <6 suppressed
SD standardized difference

© 2026 Snelgrove JW et al. *JAMA Network Open*.
