# Supplementary material for: SARS-CoV-2 Infection and Rates of Neonatal Congenital Anomalies
Source: JAMA Netw Open. 2026 May 7;9(5):e2611440. doi: 10.1001/jamanetworkopen.2026.11440 (PMC13153994; doi:10.1001/jamanetworkopen.2026.11440)
Supplement: Supplement 2. — Data Sharing Statement [file jamanetwopen-e2611440-s002.pdf]

Data Sharing Statement

Snelgrove. SARS-CoV-2 Infection and Rates of Neonatal Congenital Anomalies. *JAMA Netw Open*. Published May 07, 2026. doi:10.1001/jamanetworkopen.2026.11440

Data
Data available: No

Additional Information
Explanation for why data not available: The dataset from this study is held securely in coded form at ICES. Although data sharing agreements prohibit ICES from making the data set publicly available, access may be granted to those who meet prespecified criteria for confidential access (http://www.ices.on.ca/DAS).
